# Facial emotion recognition, affective empathy and psychosocial functioning in euthymic BD‐I

**DOI:** 10.1111/jnp.12417

**Published:** 2025-02-17

**Authors:** Susan Zyto, Nienke Jabben, Annet Nugter, Peter F. J. Schulte, Ralph W. Kupka, Sigfried Schouws

**Affiliations:** ^1^ Department of Medical Psychology Dijklander Hospital Hoorn The Netherlands; ^2^ Department of Psychiatry, Amsterdam University Medical Center Vrije Universiteit Amsterdam The Netherlands; ^3^ Department of Clinical and Medical Psychology Zuyderland Medical Centre Sittard‐Geleen The Netherlands; ^4^ Mental Health Service Organization North Holland North Alkmaar The Netherlands; ^5^ GGZinGeest Center for Mental Health Care Amsterdam The Netherlands

**Keywords:** affective empathy, bipolar disorder, emotion recognition, psychosocial functioning

## Abstract

There is emerging evidence of social cognitive impairments in bipolar disorders (BD). Less evident is the question if social cognitive impairments are predictive of psychosocial functioning, independently of neurocognitive impairment. The aims of the study were to investigate if patients with BD‐I showed impairments in facial emotion recognition and alterations in affective empathy, in relation to healthy controls, and if these impairments would predict psychosocial functioning, after accounting for neurocognitive impairments. Thirty‐seven patients diagnosed with BD‐I, in an euthymic state, and 37 matched healthy controls underwent an assessment including a facial recognition test (ERT) and a self‐report scale of affective empathy (BEES). Patients additionally underwent an extensive neuropsychological assessment consisting of traditional tests. Patients with BD‐I were significantly less able to recognize the emotion fear compared to healthy controls. However, the lower ability to recognize fear did not predict psychosocial functioning. In addition, it was not related to any of the other neuropsychological variables. The degree of self‐reported empathy did not differ between patients and healthy controls. The results add to the evidence of a specific deficit in recognizing fear in BD‐I; however, a link with psychosocial functioning was lacking. It is possible that the ability to recognize fear is related to a more narrow concept of interpersonal functioning than to the broad concept of psychosocial functioning. Future research should be directed towards aspects of social functioning in relation to social cognitive impairments, while taking account of subgroups of social cognitive functioning.

## INTRODUCTION

Bipolar disorder (BD) is a chronic psychiatric illness characterized by recurrent depressive and (hypo)manic episodes alternating with euthymic periods (American Psychiatric Association, 2013). The two major subtypes are BD type I (BD‐I) and type II (BD‐II). BD‐I is defined by episodes of mania and generally depression while in BD‐II, next to depressive episodes, only hypomanic episodes occur with less severe manic symptoms and no significant functional impairment. In both subtypes, major depressive episodes are more prevalent than (hypo)manias (Kupka et al., 2007).

BD has been described as a major cause of psychosocial dysfunction (MacQueen et al., 2001), and a considerable group of patients do not regain their premorbid level of functioning after a first hospitalization for mania (Tohen et al., 2000). Typically, patients with BD‐I have a more severe course of the illness, more hospitalizations, more impairments in daily activities and a lower employment rate (Judd et al., 2002).

In explaining impaired psychosocial functioning outcome in patients with BD, persistent neurocognitive deficits have been considered an influential factor, with studies showing small to medium associations between neurocognitive performance and outcome (Baune & Malhi, 2015; Martino et al., 2014; Wingo et al., 2009). Particularly deficits in memory and executive functioning, but also speed of processing, have been shown to predict lower psychosocial outcome (Bonnin et al., 2019; Sole et al., 2018).

Unlike neurocognitive functioning, the influence of social cognition (SC) on psychosocial outcome in BD has been less extensively studied. Social cognition refers to processes for recognition, understanding, processing and effectively using social cues for adaptive behaviour in social interactions (Harvey & Penn, 2010). Five domains have been identified to capture the concept of SC: theory of mind, emotion processing, social knowledge, social perception and attributional bias (Green et al., 2005). Assessment of social cognition typically involves making inferences about intentions and emotions from pictures, videos, faces, stories or self‐reports. Per domain specific instruments have been developed (for an overview of most common measures see de Siqueira Rotenberg et al., 2022).

A review of social cognitive deficits in patients with BD (Gillissie et al., 2022) demonstrated moderate levels of impairment in overall social cognition, while showing greater impairments in the domain of emotion processing as measured by facial emotion recognition. Patients during all mood states, as well as euthymia, have been found to misinterpret neutral faces as sad and experience problems in discriminating neutral from happy faces (Lee & Van Meter, 2020; Ruocco et al., 2014). Regarding the recognition of the particular emotions, euthymic patients with BD often fail to recognize fearful faces when compared to healthy controls (David et al., 2014; de Brito Ferreira Fernandes et al., 2016). However, the findings are mixed as other emotions have also emerged as impaired in euthymic BD, and due to heterogeneous paradigms and groups, the evidence is not easily comparable (Miskowiak et al., 2019). Furthermore, facial emotion recognition has been shown to be relatively stable over a period of 7 years (Martino et al., 2016).

In addition to impaired facial emotion recognition, BD has been associated with altered empathic responding when compared to healthy controls (Cusi et al., 2010). Empathy has been conceptualized as comprising two components, one cognitive and one affective. Cognitive empathy is the ability to make inferences about mental states of others while the affective component reflects the affective response to emotions of others (Reniers et al., 2011). While studies have frequently shown the cognitive components of empathy to be impaired in BD (Liang et al., 2020), the evidence of deficits in affective components has been more mixed. Affective empathy has in some cases been shown to be higher in BD, and can be considered a kind of over‐empathizing (Bodnar & Rybakowski, 2017), mainly associated with manic symptoms, while the cognitive components seem more linked to neurocognition (Liang et al., 2020). Other studies did not find any deficits in affective empathy in euthymic BD, and suggest it to represent areas of intact functioning (Lemvigh et al., 2022).

The relatively scarce research suggests that, in addition to neurocognition, also social cognition is associated with the level of psychosocial functioning in patients with BD. Particularly deficits in facial emotion recognition have been shown to be related to lower social and occupational outcomes (Van Rheenen & Rossell, 2014; Vlad et al., 2018). Moreover, heightened distress and lower affective empathic abilities were correlated with reduced psychosocial functioning (Cusi et al., 2010). Still, studies have yielded mixed results and it is unknown which and to what extent social cognitive impairments influence psychosocial functioning in BD, beyond neurocognitive impairments.

Given that previous suggestions that processing of socially relevant information is largely dependent on general neurocognitive functions (Bora et al., 2009a; Lahera et al., 2012) there is a need to control for the effects of neurocognition to be able to investigate if there is a unique contribution of social cognition in explaining psychosocial outcome in BD patients.

There are only few studies investigating both social cognitive and neurocognitive performance as predictors of psychosocial outcome in the same sample of bipolar patients, and results have been conflicting. Social cognitive factors have been found to mediate the relation between neurocognition and functional outcome in both BD and schizophrenia (Fett et al., 2011; Ospina et al., 2018). Other studies found that poorer verbal social cognition and higher‐level emotion processing were associated with worse psychosocial functioning, even more than neurocognitive variables (Lahera et al., 2012); although neurocognition and general social cognition were found to be strongly correlated (Aparicio et al., 2017). On the other hand, Martino et al. (2011) found no contribution of facial emotion recognition beyond neurocognition on a functional outcome measure. Inconsistencies of findings may be due to the fact that many studies used combined BD‐I and BD‐II samples, in both euthymic and symptomatic states. For that reason, it is important to examine the relative impact of social and neurocognition on outcome in a more homogeneous and clearly defined sample.

In the current study we aimed to investigate aspects of emotion processing and its relation to psychosocial outcome in a clearly defined group of euthymic patients with BD‐I, defined by an illness history of at least one clear‐cut manic episode. The aims of our study were twofold, as we investigated (i) whether BD‐I patients show impairments in facial emotion recognition and altered levels of affective empathy when compared to healthy controls, and (ii) whether impairments and alterations in these aspects of social cognition are predictive of psychosocial functioning in patients, after accounting for the presence of neurocognitive impairments.

## METHODS

### Ethical considerations

The Institutional Review Board of Mental Health Care Organization Noord‐Holland‐Noord in the Netherlands approved the study protocol. Written informed consent was obtained from all participants.

### Subjects

The study was performed in two specialized outpatient clinics for BDs, from 2013 to 2016. Data was gathered from patients with a DSM‐IV diagnosis of bipolar I disorder (BD‐I) who reported considerable subjective cognitive deficits. The patients were referred by psychologists, psychiatrists and nurses who were involved in the ongoing treatment. Patients were included when they were between 18 and 65 years old, had no severe manic or depressive symptomatology in the past 8 weeks, as defined by scores <5 on the Altman Self‐Rating Mania Scale (ASMR; Altman et al., 1997), and <16 on the Quick Inventory of Depressive Symptoms (QIDS; Rush et al., 2003). Exclusion criteria were IQ scores <85, as measured with the Dutch Adult Reading Test (DART; Schmand et al., 1998), a Dutch variant of the National Adult Reading Test, a history of neurological disease or traumatic brain injury, ECT treatment within the past 12 months, and substance or alcohol abuse within the last 3 months (defined as ≥3 units/day for men, and ≥2 units/day for women).

A control group was included to compare the social cognitive measures. The control group consisted of matched healthy participants without a diagnosis of BD‐I or BD‐II as screened by MINI (Sheehan et al., 1998), without any severe depressive symptomatology in the 8 weeks preceding the investigation (QIDS < 16), and without any current or past manic symptomatology (ASRM ≤ 5). Groups were matched by age, gender and IQ. Healthy controls were recruited among spouses of the patients, colleagues and acquaintances of assistants and main researcher.

### Instruments

#### Clinical assessment

The Questionnaire for Bipolar Illness (QBP; modified from Leverich et al., 2001) was administered in patients to gather data on sociodemographic variables, duration of illness, number of manic and depressive episodes, hospitalizations due to mania or depression, current substance or alcohol abuse and type and dose of ongoing psychotropic medication.

#### Assessment of psychosocial functioning

Psychosocial functioning in patients and controls was measured by the Functioning Assessment Short Test (FAST; Rosa et al., 2007), an interview consisting of 24 items containing six subscales representing specific aspects of daily functioning: autonomy, professional functioning, cognitive functioning, financial functioning, interpersonal relationships and free time respectively. The items were scored by the clinician on a 4‐point Likert scale consisting of: 0 = no problems, 1 = mild problems, 2 = moderate problems and 3 = severe problems. The individual scores on the six subscales were summated to achieve a total score, higher scores indicating lower levels of psychosocial functioning.

### Neuropsychological assessment

Social cognitive functioning was measured in both patients and controls. Only patients underwent an extensive assessment of neurocognitive functioning.

#### Social cognitive functioning

Two tests of *social cognition* were administered. The Balanced Emotional Empathy Scale (BEES; Mehrabian, 1996) was used as measure of affective empathy. This empathy scale consists of 15 positive and 15 negative formulated items. Each item is answered by the subject on a 9‐point scale that runs from −4 (strongly disagree) to +4 (strongly agree). The total score is calculated by summing up the responses to all negatively formulated items and subtracting the response of all positively worded items. Higher scores indicate a greater predisposition to experience other people's emotions.

The Emotion Recognition Task (ERT; Montagne et al., 2007) was administered to assess facial emotional perception. The ERT consists of video clips of four faces (two male and two female) that gradually express six emotions, namely angry, happy, sad, scared, surprised and disgusted. In total, the video clips consisting of four blocks of 24 trials are shown. For every recognized emotion a point is given.

#### Neurocognitive functioning

*Pre‐morbid intelligence* was estimated through the DART (Schmand et al., 1998). *Processing speed* was measured with three tests: the Digit Symbol Substitution Test (DSST), subtest of the Wechsler Adult Intelligence Scale IV (WAIS‐IV‐NL; Wechsler, 2008), the Trail Making Test (TMT; Reitan, 1958) part A and the colour naming and colour word reading cards of the Stroop Colour Word Test (SCWT; Hammes, 1971). *Working memory* was assessed by the Digit span subtest of the WAIS‐IV‐NL (Wechsler, 2008). *Verbal memory* was assessed by a Dutch variant of the Rey's Auditory Verbal Learning Task (the 15‐words test; Saan & Deelman, 1986), consisting of an encoding condition of five trials, delayed reproduction and recognition. *Executive functioning and mental flexibility* was measured by the Wisconsin Card Sorting Test (WCST; Heaton, 1981): the number of completed categories and the amount of perseverative errors where recorded. As measures of *verbal fluency*, category fluency as well as the letter fluency test were conducted (Bouma et al., 2012). Three separate total scores were calculated, two for category fluency (animals and professions respectively) and one for letter fluency.

### Data analysis

Statistical Package for Social Sciences (SPSS) version 27 (IBM Corp., Armonk, NY, USA) was used to conduct the statistical analyses. Significance was defined by *p*‐values <.05. Quantitative variables were described using the mean and standard deviation. Ordinal data were presented by median and range. Data were checked for normality. For non‐normally distributed data the median and interquartile range were used.

To examine differences between the patients and controls on social cognitive measures, independent *t*‐tests were performed. For non‐normally distributed variables a non‐parametric test was used, the Mann–Whitney *U*‐test. In order to analyse difference in sex distribution a chi‐square test was performed.

We controlled for significant differences in level of education between the patients' group and the healthy controls by a linear regression for each of the emotions and empathy as a dependent variable and education along with group as an independent variable.

To examine if SC measures modulate psychosocial outcome in the patients' group, we performed a linear regression analysis with psychosocial functioning as the dependent variable and SC measures along with group as independent variable. To investigate the impact of social cognition on psychosocial functioning, first Pearson correlation coefficients were used to investigate the relation between social cognitive, neurocognitive, clinical and demographic variables. Then, a hierarchical regression was performed to explore the dependence of social cognitive impairments on psychosocial functioning while controlling for clinical, demographic and neurocognitive variables. Only neurocognitive variables that deviated more than a *z*‐score of −1, compared to controls, were included.

Neurocognitive variables were compared with matched data from controls drawn from the normative database ANDI (de Vent et al., 2016). ANDI is a normative database developed by the University of Amsterdam for clinical as well as research purposes. It comprises test results from the most commonly used neuropsychological tests and were gathered from more than 26,000 healthy controls. *Z*‐scores were calculated to represent deviations of patients relative to controls.

## RESULTS

### Clinical and sociodemographic characteristics

In total 37 patients with BD‐I and 37 healthy controls participated in the study. For demographic and clinical characteristics of the patients and the controls, see Table 1. Patients had a significantly lower level of education and higher degree of (residual) depressive symptoms than controls. Psychosocial functioning was significantly lower in the patient group.

**TABLE 1:** Demographics and clinical characteristics.

Characteristics	Patients (*n* = 37)	Controls (*n* = 37)	Statistics	*p*‐Value
	Mean (SD)	Mean (SD)	*t*(*df*)/*U*/*χ* ^2^	
Age	48.1 (10.4)	45.3 (13.5)	*t*(72) = −.98	.33
Gender (% female)	62.2	62.2	*χ* ^2^(1) = .00	1
Educational level^a^ (*Mann Whitney U‐test*)	Median 6 Range 2–7	Median 6 Range 5–7	*U* = 505.0	.028*
Estimated IQ (DART)	102.4 (10.0)	102.4 (8.7)	*t*(70) = .022	.98
Duration of illness (years)	23.8 (11.0)	–		
Manic episodes (number)	4.8 (3.4)	–		
Depressive episodes (number)	4.9 (3.6)	–		
Hospitalizations (number)	1.6 (2.1)	–		
Depressive symptoms (QIDS)	8.7 (4.6)	2.9 (2.3)	*t*(72) = −6.88	<.001*
Manic symptoms (ASRM)	2.4 (3.5)	2.0 (2.4)	*t*(72) = −.50	.62
Psychosocial functioning (FAST)	27.3 (8.6)	.9 (1.4)	*t*(72) = −18.4	<.001*

Abbreviations: ASRM, Altman Self‐Rating Mania Scale; DART, Dutch Adult Reading Test; FAST, Functional Assessment Short Test, higher scores indicate more problems; QIDS‐SR, Quick Inventory of Depressive Symptomatology‐Self‐Rated.

^a^ Education is based on Dutch education scores 1–7 (Verhage 1964), corresponding with the following US years of education: 1; 1–5 years, 2: 6 years, 3: 7–8 years, 4: 7–9 years, 5: 7–10 years, 6: 7–16 years and 7: 17–20 years.

* *p*‐Values <.05 are considered significant.

### Social cognitive performance

The social cognitive test performance of patients and controls are presented in Table 2. No significant differences between patients and controls were found on the total emotion recognition score. When investigating individual emotions, only fear recognition differed significantly between patients and controls, patients demonstrating lower recognition of fear. There was no significant difference between patients and controls on self‐reported empathy. Controlling for different levels of education between groups did not alter the results.

**TABLE 2:** Social cognitive functioning.

Characteristics	Patients (*n* = 37)	Controls (*n* = 37)	Statistics	*p*‐Value	*z*‐scores
	M(SD)/Mdn(IQR)	M(SD)/Mdn(IQR)	*t(df)*/*U*		
ERT total M(SD)	58.27 (9.80)	62.14 (7.19)	*t*(72) = −1.94	.06	−.54
ERT surprise Mdn(IQR)	9 (3)	9 (4)	*U* = 668.5	.86	−.05
ERT sadness M(SD)	6.78 (3.23)	8.03 (2.68)	*t*(72) = −1.80	.09	−.47
ERT anger Mdn(IQR)	11 (4)	14 (3)	*U* = 592.5	.31	.05
ERT fear Mdn(IQR)	4 (5)	7 (4)	*U* = −402.5	.002*	−.81
ERT disgust Mdn(IQR)	11 (6)	11 (4)	*U* = 613.5	.44	−.20
ERT happiness Mdn(IQR)	15 (1)	15 (1)	*U* = 614.0	.43	−.13
BEES M(SD)	26.57 (30.81)	29.35 (24.22)	*t*(72) = −.43	.67	−.11

Abbreviations: BEES, Balanced Emotional Empathy Scale; ERT, Emotion Recognition Task; IQR, interquartile range; M, mean; Mdn, median.

* Statistically significant at a < .05 level.

### Neurocognitive assessment in patients

Results from the neuropsychological assessment in patients and *z*‐scores as compared to a normative group of healthy controls, matched on age, education and gender, drawn from the database ANDI (de Vent et al., 2016), are shown in Table 3. The greatest deviations from controls (defined by *z*‐scores <−1) were found in processing speed and attention (working memory).

**TABLE 3:** Neurocognitive performance in patients (*n* = 37).

	Mean (SD)	Range	*z*‐scores (SD)
Processing speed			
DSST (WAIS‐IV)	61.5 (14.0)	38–86	−.70 (.87)
TMT‐A	46.6 (17.8)	19–100	−1.2 (1.0)
SCWT‐word	49.4 (10.7)	35–74	−.86 (1.2)
SCWT‐colour	66.8 (12.8)	45–98	−1.1 (1.2)
SCWT‐word/colour	99.5 (24.2)	61–180	−.38 (1.0)
Executive functions			
WCST cat	4.60 (1.9)	0–6	−.32 (1.1)
WCST pers. errors	15.9 (13.2)	0–41	.58 (1.4)
Fluency animals	24.0 (6.2)	13–41	−.29 (.94)
Fluency occupations	16.7 (5.00)	6–29	−.49 (.98)
Fluency phonological	40.5 (10.6)	20–61	−.05 (1.3)
TMT B	90.4 (32.4)	53–109	−.80 (.84)
Attention			
Digit Span (WAIS‐IV)	23.5 (5.27)	15–35	−1.1 (1.2)
Memory			
15wt immediate recall	40.4 (13.57)	16–63	−.77 (1.3)
15wt delayed recall	8.2 (3.87)	1–15	−.70 (1.3)
15wt recognition (total 30)	28.0 (2.20)	23–30	−.60 (1.0)

Abbreviations: 15‐WT, 15 Woorden test (Dutch variant of California Verbal Learning Test); DAT, phonemic fluency, animal naming: categorical fluency; DSST, Digit Symbol Substitution Test from Wechsler Adult Intelligence Scale‐IV (WAIS‐IV); SCWT, Stroop Color Word Test (Interference); TMT, Trail Making Test; WCST, Wisconsin Card Sorting Test.

### Relationship between social cognition and psychosocial functioning

To analyse if SC measures modulate psychosocial outcome, we performed a linear regression analysis with FAST as the dependent variable and the social cognitive measures along with group as independent variable. No significant effect was found.

Correlation analyses showed that social cognitive tasks did not correlate with any of the demographic or clinical variables such as age, IQ, current mood, chronicity, number of hospitalizations, number of depressive episodes or manias. Likewise, social cognitive tasks were not significantly correlated with any of the neurocognitive variables.

We performed a hierarchical regression with FAST total score as dependent variable and age, depressive symptoms, TMT‐A, Stroop Colour and Digit Span in the first block and fear recognition in the last block. Only age was significantly associated with FAST score (ß = .459; *p* = .002).

An analysis of the fear variable in bipolar patients revealed a wide spread of scores, ranging from 0 to 12, and a substantial heterogeneity (SD = 3.2). To account for this variability, two groups were created by performing a median split: a low‐performing (LP) group (scores ≤4; 51.2%, *n* = 19; m = 2.11; SD = 1.33) and a high‐performing (HP) group (scores >4: 48.8%, *n* = 18; m = 7.17; SD = 2.53). This division allowed for the exploration of potential differences between the groups.

Repeating the regression with the LP/HP fear recognition subgroups revealed no differences between these groups in their associations with FAST as a dependent variable.

To further investigate if there were any differences between the LP and HP group in clinical, demographic, neurocognitive and social cognitive variables, additional analyses were performed. The results show that the LP and the HP group did not differ on any of the clinical or demographic variables, nor any of the neurocognitive variables.

However, the LP group occurred to have a significantly lower ability to recognize sadness (*U* = 104; *p* = .04) compared to the HP group. When performing a regression with FAST as a dependent variable and LP/HP group and fear and sadness, age, depressive symptoms, TMT‐A, Stroop Colour and Digit Span as independent variables no significant differences between the groups occurred. Again, only age was a significant predictor of psychosocial functioning (ß = .502; *p* = .002).

## DISCUSSION

In the current study, we aimed to investigate facial emotion recognition and affective empathy in a clearly defined sample of euthymic patients with BD‐I, and to analyse whether social cognitive impairments would predict psychosocial functioning, while accounting for neurocognitive performance.

The results show that BD‐I patients demonstrated a significantly lower ability to recognize fear in a facial emotion recognition task, compared to healthy controls. However, this lower ability was not associated with the psychosocial functioning in patients. In addition, no associations between fear recognition and psychosocial functioning were found in the subgroup of patients with the lowest ability (LP) to recognize fear. Moreover, the ability to recognize fear, was not correlated with any of the neurocognitive or clinical variables in BD patients, as well as in the LP subgroup. Furthermore, self‐rated affective empathy did not differ between patients and controls, nor was there an association with outcome.

The current findings of a reduced fear recognition in euthymic BD‐I patients is in line with previous studies of social cognition in BD (Gillissie et al., 2022) that reported stable deficits in fear recognition. It can be hypothesized that impaired fear recognition is a risk factor for the development of mania since perception of fear should act as an inhibition of risk‐seeking behaviour associated with mania. It has been suggested that intact emotion recognition is necessary for appropriate self‐regulation (Fulford et al., 2014) and that the ability to recognize fear in others correlates positively with one's own experience of fear (Buchanan et al., 2010). Therefore, a reduced ability to recognize fear may hinder adequate feedback on risky behaviour during (hypo) mania, thereby increasing the risk of further manic behaviour through inadequate feedback loops. Research demonstrating that the 6–14‐year‐old offspring of parents with BD‐I show similar deficits in the recognition of fear (Sharma et al., 2017) further underscores the idea that diminished fear recognition may be a risk factor for BD‐I. However, in the current study the number of manic episodes was not associated with the ability to recognize fear, as hypothetically, lack of self‐regulation could manifest itself in increased episodes of mania.

Although in this study a comprehensive neuropsychological assessment was included to be able to control for the influence of neurocognitive performance on social cognitive functioning and outcome, no correlations between emotion recognition and neurocognitive measures were found. Previous studies on the relationship between fear recognition ability and neurocognitive functioning yielded mixed results. Similar to the current study, Szmulewicz and colleagues (2019) found no correlations between general cognition and the recognition of fear, while Martino et al. (2011) demonstrated that lower fear recognition was associated with lower verbal memory, attention and executive functioning, but not with any of the clinical variables. These differences can be due to characteristics of the study group as almost all of the BD‐I patients in the Martino study had a psychosis in the past, which is known to be associated with more severe neurocognitive impairments (Bowie et al., 2018).

Despite the lower fear recognition in BD‐I patients in our study, we failed to find an association with psychosocial functioning. This is in line with two previous studies (Martino et al., 2011; Szmulewicz et al., 2019) that both failed to find an association between lower fear recognition and outcome. An initially found relationship between recognition of fearful expression and psychosocial functioning disappeared after controlling for neurocognitive variables, suggesting there was no additional contribution from SC on outcome (Martino et al., 2011). Contrary, one previous study (Fulford et al., 2014) found that a better ability to recognize fearful faces was related to a higher quality of life after accounting for demographic and mood variables, but in that study the influence of general cognition was not taken into account.

Previous studies on neurocognitive performance in BD showed that patients can be clustered in at least three subgroups with different neurocognitive performance profiles: one of which is cognitively unimpaired while the other two show neurocognitive profiles with selective or global impairment respectively (Burdick et al., 2014). More recently, similar findings in social cognition have been found, with about two‐thirds of BD patients showing intact social cognition and one‐third demonstrating mild SC deficits (Varo et al., 2020). Based on these findings we divided the current sample in a high‐ and low‐performance group on the fear recognition variable to exploratively analyse differences between the subgroups. However, high‐ and low‐scoring patients did not differ in their association with psychosocial outcome in the current sample.

Earlier findings of altered affective empathy in patients with BD compared to healthy controls (Bodnar & Rybakowski, 2017; Cusi et al., 2010) could not be replicated in this study. This may be due to the inclusion of a strictly defined euthymic BD‐I sample, as altered affective empathy has most often been found in association with actual manic symptoms (Liang et al., 2020). Most studies in euthymic BD‐I did not show differences in affective empathy which is suggested to be a marker of resilience (Lemvigh et al., 2022). Even though Cusi et al. (2010) found a correlation between affective empathy and social functioning, no significant differences has been found in this variable in comparison with healthy controls. It should be noted, however, that affective empathy typically is measured by self‐report questionnaires as it was in our study. As such, it is dependent on the responder's insight into their own behaviour and is also more prone to socially desirable questionnaire completion.

In explaining the conflicting results between different studies of social cognition in BD, several factors should be taken into account. Many of the measurements for SC were developed for the study of autism spectrum disorders and schizophrenia, but none were especially developed or validated for BD and there is little consensus on which tests should be used. In addition, the tests themselves have been poorly validated and the psychometric properties are not always clear. Furthermore, studies have used a heterogeneity of tools and measures for social cognition making direct comparisons more difficult. Additionally, various studies have included patients in different mood states, and even in euthymic states residual depressive symptoms can influence social cognition. Also, findings that within BD‐I subgroups with various neurocognition and social cognition performance profiles have been described (Burdick et al., 2014; Varo et al., 2020) may add to the contrasting findings.

In addition to the more general considerations about the measurement of social cognition, our study has several limitations. As previously mentioned, a limitation of the current study is that measurement of empathy was based only on a self‐report questionnaire. Perhaps an additional measure by a proxy could give more validity to the general measurement of empathy. Furthermore, the limited sample size prevented further and more reliable subgroup analyses. Another limitation comes from the fact that we didn't take into account psychiatric medication, which may be relevant since pharmacological treatment has been associated with reduced social cognition (Szmulewicz et al., 2019).

The strength of the current study lies in examining both social cognition and a comprehensive battery of neurocognitive measures in the same, clearly defined bipolar sample. This is important because these variables have been shown to be highly correlated (Bora et al., 2009b; Lahera et al., 2012). Nevertheless, we did not find a correlation between the ability to recognize fear and neurocognitive variables. It is important to notice, however, that many of the studies of social cognition included verbal social cognitive measures and measures of general emotional intelligence which might rely more heavily on general neurocognition for information processing, and this might apply less for emotion recognition.

We recommend that future research should define the groups more sharply according to subgroups of social cognitive functioning and investigate to what extent the social and neurocognitive variables are predictive of the psychosocial functioning in each subgroup. It is not yet clear in what way the established social cognition deficits influence outcome in BD patients. It could be fruitful to investigate further if the deficits in social cognition could perhaps be related to a more narrow concept of interpersonal functioning rather than a broad measure of psychosocial outcome. In addition, the hypothesis that impairments in fear recognition can act as a risk factor for developing mania should be further investigated.

## CONCLUSION

This study of 37 euthymic patients with BD‐I showed that patients had a significant lower ability to recognize fear in a facial emotion recognition task, in comparison with healthy controls, independent of neurocognitive and clinical variables. A lower ability to recognize fear was not predictive of psychosocial functioning, even in a subgroup scoring lowest on fear recognition. It may be of clinical significance if failing to recognize fear in others and self may increase the risk for risk‐seeking behaviour and manic disinhibition.

## AUTHOR CONTRIBUTIONS

**Susan Zyto:** Conceptualization; methodology; investigation; formal analysis; writing – original draft. **Nienke Jabben:** Conceptualization; methodology; writing – original draft. **Ralph W. Kupka:** Conceptualization; methodology; writing – review and editing. **Annet Nugter:** Writing – review and editing. **Peter F. J. Schulte:** Writing – review and editing. **Sigfried Schouws:** Writing – review and editing.

## CONFLICT OF INTEREST STATEMENT

Authors have no conflicts of interest to declare.

## Data Availability

Data are available upon reasonable request.
